# Evaluation of neuropsychiatric comorbidities and their clinical characteristics in Chinese children with asthma using the MINI kid tool

**DOI:** 10.1186/s12887-019-1834-7

**Published:** 2019-11-22

**Authors:** Hao Zhou, Zhihe Chen, Weiqing Zhao, Ye Liu, Yuxia Cui

**Affiliations:** 1 Department of Pediatrics, Guizhou Provincial People’s Hospital, Medical College of Guizhou University, No. 83, Zhongshan Road, Nanming District, Guiyang, 550002 China; 2 Otolaryngological Department, Guizhou Provincial People’s Hospital, Medical College of Guizhou University, Guiyang, China

**Keywords:** Asthma, Comorbidity, Risk factors, Children

## Abstract

**Background:**

The mental health and quality of life in children with asthma have attracted widespread attention. This study focused on the evaluation of mental health conditions and their clinical characteristics in Chinese children with asthma.

**Methods:**

A total of 261 children with asthma aged 6 to 16 years old and 261 age- and gender-matched children from the general population were recruited to participate in this study from Guizhou Provincial People’s Hospital. The parents of all subjects were interviewed using the MINI Kid and were required to finish a clinical characteristics questionnaire. Logistic regression analysis was performed to evaluate risk factors.

**Results:**

The prevalence of mental health conditions in the asthma group was significantly higher than that in the control group (26.4% vs 14.6%, *P < 0.001*). A total of 10 mental health conditions was identified in the asthma group, the most common of which was ADHD (11.5%; 30/261), followed by oppositional defiant disorder (ODD) (10.7%; 28/261), separation anxiety disorder (6.1%; 16/261), social anxiety disorder (3.8%; 10/261), specific phobias (2.3%; 6/261), agoraphobia without panic (1.5%; 4/261), (mild) manic episodes (1.1%; 3/261), major depressive episodes (MDEs) (0.8%; 2/261), movement (tic) disorder (0.8%; 2/261), and dysthymia (0.4%; 1/261). A total of 6 neuropsychiatric conditions was detected in the control group, including ODD (5.7%; 15/261), ADHD (4.6%; 12/261), social anxiety disorder (3.1%; 8/261), seasonal anxiety disorder (SAD) (2.3%; 6/261), specific phobias (1.1%; 3/261), and agoraphobia without panic (0.4%; 1/261). The prevalence rates of ODD, ADHD, and SAD differed significantly between the two groups (*P* < 0.05). Multiple regression analysis revealed that severe persistent asthma (OR = 3.077, 95% CI 1.286–7.361), poor asthma control (OR = 2.005, 95% CI 1.111–3.619), and having asthma for > 3 years (OR = 2.948, 95% CI 1.580–5.502) were independent risk factors for the presence of mental health conditions in asthmatic children.

**Conclusions:**

Children with asthma have a higher rate of mental health conditions than non-asthmatic children. Standardized diagnosis and treatment may help reduce the risk of neuropsychiatric conditions.

## Background

Chronic diseases are public health problems that affect population health, but previous studies have mainly focused on chronic diseases in adults. Currently, experts are paying more attention to the mental health of children with chronic diseases. The prevalence of chronic diseases in children has been reported to range from 11 to 56.7%, and the incidence of mental health disorders in children with chronic conditions has been reported to range from 13.8 to 62% [1, 2]. Of note, chronic diseases lasting more than 3 years may be associated with a higher risk of neuropsychiatric comorbidities than chronic diseases with a duration of less than 3 years [3]. The economic burden of mental health conditions in children with asthma has reached 10.9 billion dollars in the United States [2], and considerable economic and disease burdens have been placed upon both families and societies [4].

Several mental health conditions, including anxiety, depression, social withdrawal and other psychological and behavioural disorders, have been found to be common in Western populations and are commonly associated with chronic diseases such as asthma due to their associated medical treatments and long-term disease duration [5–7]. When these problems cannot be identified and resolved in a timely manner, they often continue into adulthood and may even last throughout life, resulting in more negative effects, such as unemployment, substance abuse, and criminal behaviour, bringing about even more social problems [8, 9]. However, the prevalence of and risk factors for different mental health conditions in Chinese children with chronic disease patients remain unclear, and further research is needed. Asthma is one of the most commonly occurring chronic diseases in children, with approximately 300 million people living with asthma worldwide, and the rates of morbidity and mortality associated with this condition are substantial [10]. The prevalence of asthma in urban children has reached 3.02% in China [11], increasing steadily over the last few decades. Thus, asthma has become a global public health concern.

In recent years, the medical field has paid more attention to the physical and mental health of children with asthma. In 2000, a French study surveyed the prevalence of health conditions in children with asthma and found that the total prevalence rate of mental health conditions was 42% [12]. The results of a US study conducted in 2002 [13] showed that the total prevalence rate of mental health conditions in children with asthma was 56.3%. In contrast, a Norwegian longitudinal study of school children aged 8 to 10 showed that the overall prevalence of mental health conditions was relatively low (7 to 20%) [14]. The prevalence of mental health conditions in children with asthma has been reported to range from 25 to 63% [15–21]. In a large cohort of Swedish twins (*N* = 20,072), children with asthma had a two-fold higher risk of ADHD than children without asthma [17]. Another study found clinically relevant anxiety levels in 41% of asthma patients compared to only 17% of non-asthmatic control patients [15]. In 2004, a longitudinal birth cohort study [6], among community youth followed until age 21, found that adolescents and young adults with asthma had an increased likelihood to develop any type of anxiety disorder and major depressive syndrome. A recent study by Wu et al. [22] found an association between both maternal and paternal asthma and bipolar disorder, but no association was found between parental asthma and schizophrenia spectrum disorders.

Mechanisms that may help to explain associations between asthma and mental health are not yet well understood but may include asthma-related impaired social or physical functioning, drug effects, underlying disease characteristics such as inflammatory response or the dysregulation of neuroendocrine activity, and/or the experience of unstable asthma symptoms [21–24]. Goodwin et al. [23] suggested that possible biological and psychological mechanisms may include inflammatory processes along with the stress of living with a life-threatening illness. It has also been suggested that mental health status may increase the risk of asthma [25]. For example, the US CARDIA study, a 20-year longitudinal study, found that depression was a risk factor for developing asthma [26]. Similarly, a study from Korea found that ADHD patients had a higher risk of asthma than non-ADHD patients [16]. However, the temporal link between mental health and asthma remains unclear [16, 27].

The high prevalence of comorbid mental health disorders has a more substantial impact on children, family and society than asthma itself. Until now, few studies have been conducted on mental health conditions in Chinese children with asthma. One study showed that evaluating mental health using mental health assessment scales revealed no association between asthma and depressive symptoms in a Chinese population (*N* = 9280) [21]. However, a large cohort study conducted in Taiwan (*N* = 30,169) found that adults with major depressive disorder (MDD) were at a 1.66 higher risk of asthma than non-depressive adults [27]. The present study focused on the prevalence of and risk factors for mental health conditions in Chinese children with asthma.

## Methods

### Study population

The present study was conducted from January 1, 2015, to December 31, 2016, and the study sample was divided into two groups: an asthma group and a non-asthmatic control group. Guizhou Provincial People’s Hospital is located in the city of Guiyang, which has a population of approximately 4.6 million, and is considered the premier hospital in the region. Guizhou is a relatively poor and economically undeveloped province. The study was performed in accordance with the Declaration of Helsinki, and all parents/guardians gave their written informed consent.

### Asthma group

We recruited 6 to 16 year-old children with asthma from the Children’s Asthma Outpatient Department of Guizhou Provincial People’s Hospital in China. Criteria for children with asthma included: (1) diagnosis of asthma according to the Guidelines for the Management and Prevention of Asthma in Children [28]; (2) no other chronic disease, such as diabetes, epilepsy, migraines, or tumour; and (3) no family history of psychosis. The exclusion criteria included the following: (1) children with asthma comorbid with other chronic diseases (diabetes, epilepsy, migraines, and tumour, etc.); and (2) refusal of the parents of the children to participate.

The diagnostic standards for asthma were based on the Bronchial Asthma Diagnostic and Prevention Guide for Children (2016 version). The diagnosis of asthma was performed by specialists with the title of chief physician in the Children’s Asthma Outpatient Department at Guizhou Provincial Peoples’ Hospital; all diagnosing specialists had at least 10 years of familiarity with asthma diagnosis.

### Control group

The control group consisted of age- and gender-matched children with typical development who visited the health examination centre of the hospital for annual routine outpatient health exams provided for all school-age children in China. Children who had chronic diseases, such as asthma, diabetes, epilepsy, or migraines, were excluded from the study. Children with transient emotional distress due to hospital visits were not diagnosed as having mental health difficulties unless defined as manic episodes, as previously described [29].

### Sample size

Sample size calculations were performed, with an attention deficit hyperactivity disorder (ADHD) prevalence of 14.3 and 7.1% in children with asthma and typical development children, respectively, according to our previous study [30]; because ADHD is one of the most common mental health conditions in children, an alpha of 0.05, a power of at least 0.8, and a match ratio of 1:1 were needed. Based on this calculation with Power and Sample Size Calculation Setup, approximately 288 subjects were needed for this study.

### MINI kid tool

The Mini-International Neuropsychiatric Interview for children and adolescents (MINI Kid) is an interactive interview tool developed by Profs. Sheehan and Lecrubier to serve as an effective and reliable evaluation of neuropsychiatric disease in young people [31]. The items included in this tool are mainly derived from the diagnostic criteria for a variety of neuropsychiatric diseases included in the Diagnostic and Statistical Manual of Mental Disorders, 4th edition (DSM-IV) and International Classification of Diseases, 10th Revision (ICD-10). The tool is considered suitable for children aged 6–16 years old [31]. The interview was divided into components for children and parents and included questions regarding 23 mental disorders; each part included screening questions and diagnostic questions. During the interviews, the investigator, who had received training on the MINI-kid interview questionnaire, read the question, and the parents answered “yes” or “no” so that a final diagnosis could be assigned. Each interview took approximately 30 to 40 min. In addition, the investigators who administered the test were attending physicians and had at least 5 years of experience in the diagnosis of neuropsychiatric illnesses in children. At present, the scale has been translated into multiple languages and is widely used internationally in a variety of epidemiological neuropsychiatric comorbidity studies [32].

The Chinese version of the MINI Kid was developed for parents and children by Peking University Sixth People’s Hospital and has been found to have good reliability and validity [33]. In the present study, the parental version of the MINI Kid was used to investigate the rate of neuropsychiatric comorbidities in asthmatic children with the approval of the author of the Chinese version. Because the focus of the present study was whether children with asthma were more likely than children in the general population to have mental disorders, clinical characteristics of asthma-related mental health were defined according to the Global Initiative for Asthma guidelines [28].

### Questionnaire addressing demographics and asthma clinical characteristics questionnaire

To study the risk factors for neuropsychiatric comorbidities, a questionnaire including the basic demographics of the patients and clinical characteristics was designed by the authors (see the Additional file 1), with the basic demographics including gender, age, place of residence, ethnicity, family income, and education level of the primary caregiver. The asthma clinical characteristics questionnaire was designed based on the Global Initiative for Asthma guidelines and was intended to collect data on the first onset of asthma, severity of the disease, level of control, duration of disease, regularity of physician visits, frequency of onset and drug use, family history of asthma, and other issues. The criteria of asthma severity and control were based on the Asthma Management and Prevention Guidelines, interpreted for prevention and treatment in Chinese children [28]. The questionnaire was not evaluated for reliability and was not validated; therefore, psychometric properties were not available.

### Study procedure

The parents who gave written informed consent for their children to participate were invited to participate in this research. All parents underwent a face-to-face MINI Kid questionnaire interview and to complete the basic demographics. In addition, the parents of children with asthma received a face-to-face asthma clinical characteristics questionnaire interview by a trained child physician in the hospital.

### Statistical analysis

Statistical analysis was performed using Stata 11.0 software (version 11.0, College Station, Texas 77,845, United States). Descriptive statistical methods were generated for the patient characteristics; continuous variables were analysed using independent *t*-tests, and categorical variables were analysed using chi-squared or Fisher’s exact tests. To explore the association between asthma-related risk factors and children with comorbid neuropsychiatric diseases, we first employed univariate logistic regression analysis. Then, according to univariate analysis results, we selected four factors that demonstrated significant differences for multivariate logistic regression. The four factors included the severity of illness, level of disease control, duration of disease, and asthma drug use. All tests were two-tailed, and the significance level was set at α = 0.05.

## Results

### Basic demographic characteristics

From January 1, 2015, to December 31, 2016, 306 children (6–16 years old) with asthma who were outpatients at the hospital were included in this study, and 85.3% (261/306) of the parents of the asthmatic children who provided written informed consent were then invited to participate in this research. The other 14.7% of parents of the asthmatic patients refused to give consent. A total of 261 children with asthma, including 160 males (61.3%), with a mean age of 9.35 ± 2.52 years and a total of 261 control children, including 162 males (62.0%), with a mean age of 9.08 ± 2.43 years were recruited to participate in this study. No significant differences in sex and age were identified between the two groups (both *P > 0.05*). Detailed demographic characteristics are described in Table 1.

**Table 1:** Demographic characteristics of asthma and control group children

Characteristics	Category	Asthma group (*n* = 261)	Control group (*n* = 261)	χ^2^/t	*P*
Age		9.35 ± 2.52	9.08 ± 2.43	0.237	0.813
Place of residence	Urban	154(59.0)	162(62.0)	0.513	0.474
	Suburban or rural	107(41.0)	99(38.0)		
Ethnicity	Han	210(80.5)	195(74.7)	2.479	0.115
	Ethnic minority	51(19.5)	66(25.3)		
Educational level of chief caregiver	High school and above	150(57.5)	134(51.3)	1.977	0.160
	Below high school	111(42.5)	127(48.7)		
Monthly household income (RMB)	<2000/month	45(17.2)	52(19.9)	1.754	0.416
	2000–5000/month	101(38.7)	87(33.3)		
	>5000/month	115(44.1)	122(46.7)		
Only child	Yes	148 (56.7)	161 (61.7)	1.34	0.247
	No	113 (43.3)	100 (38.3)		
Left-behind child	Yes	21 (8.1)	13 (5.0)	2.014	0.156
	No	240 (91.9)	248 (95.0)		
Parental relationship	Harmonious	215 (82.4)	230 (88.1)	3.428	0.064
	Not harmonious	46 (17.6)	31 (11.9)		
Family structure	Single-parent family	11 (4.2)	8 (3.1)	2.986	0.225
	Two-parent family	170 (65.1)	155 (59.4)		
	Three generations	80 (30.7)	98 (37.5)		
Family history of psychiatric disease	Yes	3 (1.1)	1 (0.4)	1.008	0.315
	No	258 (98.9)	260 (99.6)		
Child-raising method	Authoritative	193 (73.9)	208 (79.7)	5.249	0.154
	Arbitrary	64 (24.5)	45 (17.2)		
	Permissive	3 (1.1)	6 (2.3)		
	Negligent	1 (0.4)	2 (0.8)		
Living in school	Yes	46 (17.6)	32 (12.3)	2.954	0.086
	No	215 (82.4)	229 (87.7)		
Health insurance	Yes	201 (77.0)	212 (81.2)	1.403	0.236
	No	60 (23.0)	49 (18.8)		

Continuous variables are shown as the mean ± standard deviation; categorical variables are shown as counts (%)

### Prevalence of neuropsychiatric comorbidities in asthma and control group children

A total of 69 asthma group children had neuropsychiatric comorbidities, with a prevalence rate of 26.4% (69/261). The neuropsychiatric comorbidity rate was slightly higher in females than in males (29.7% vs 24.4%), but the difference was not significant. A total of 38 children in the control group had neuropsychiatric comorbidity, with a prevalence of 14.6% (38/261). The difference in comorbidity rates between the asthma and control groups was significant (*P* < 0.05). A total of 10 mental health conditions were identified in the asthma group, the most common of which was ADHD (11.5%; 30/261), followed by oppositional defiant disorder (ODD) (10.7%; 28/261), separation anxiety disorder (6.1%; 16/261), social anxiety disorder (3.8%; 10/261), specific phobias (2.3%; 6/261), agoraphobia without panic (1.5%; 4/261), (mild) manic episodes (1.1%; 3/261), major depressive episodes (MDE) (0.8%; 2/261), movement (tic) disorder (0.8%; 2/261), and dysthymia (0.4%; 1/261). A total of 6 neuropsychiatric conditions were detected in the control group, including ODD (5.7%; 15/261), ADHD (4.6%; 12/261), social anxiety disorder (3.1%; 8/261), seasonal anxiety disorder (SAD) (2.3%; 6/261), specific phobias (1.1%; 3/261), and agoraphobia without panic (0.4%; 1/261). The prevalence rates of ODD, ADHD, and SAD differed significantly between the two groups (*P* < 0.05) (Table 2).

**Table 2:** Distribution of neuropsychiatric comorbidities in the asthma and control groups (n, %)

Psychiatric comorbidities	Asthma group (*n* = 261)	Control group (*n* = 261)	χ^2^	*P*
Attention deficit hyperactivity disorder	30(11.5)	12(4.6)	8.389	0.004
Inattentive type	18(6.9)	8(3.1)		
Combined type	9(3.4)	3(1.1)		
Hyperactive impulsive	3(1.1)	1(0.4)		
Oppositional defiant disorder	28(10.7)	15(5.7)	4.283	0.038
Separation anxiety disorder	16(6.1)	6(2.3)	4.745	0.029
Social anxiety disorder	10(3.8)	8(3.1)	0.230	0.631
Specific phobia	6(2.3)	3(1.1)	0.452	0.501^a^
Agoraphobia without panic	4(1.5)	1(0.4)	0.808	0.369^a^
(Mild) manic episodes^c^	3(1.1)	0(0.0)		0.249^b^
Major depressive episode	2(0.8)	0(0.0)		0.499^b^
Tic disorder	2(0.8)	0(0.0)		0.499^b^
Dysthymia	1(0.4)	0(0.0)		1.000^b^

^a^continuous correction chi-squared test, ^b^Fisher’s exact test, ^c^A manic episode is defined in the American Psychiatric Association’s Diagnostic Manual (DSM-5) as a “distinct period of abnormally and persistently elevated, expansive, or irritable mood and abnormally and persistently increased activity or energy, lasting at least 1 week and present most of the day, nearly every day (or any duration if hospitalization is necessary)”

**Fig. 1:**
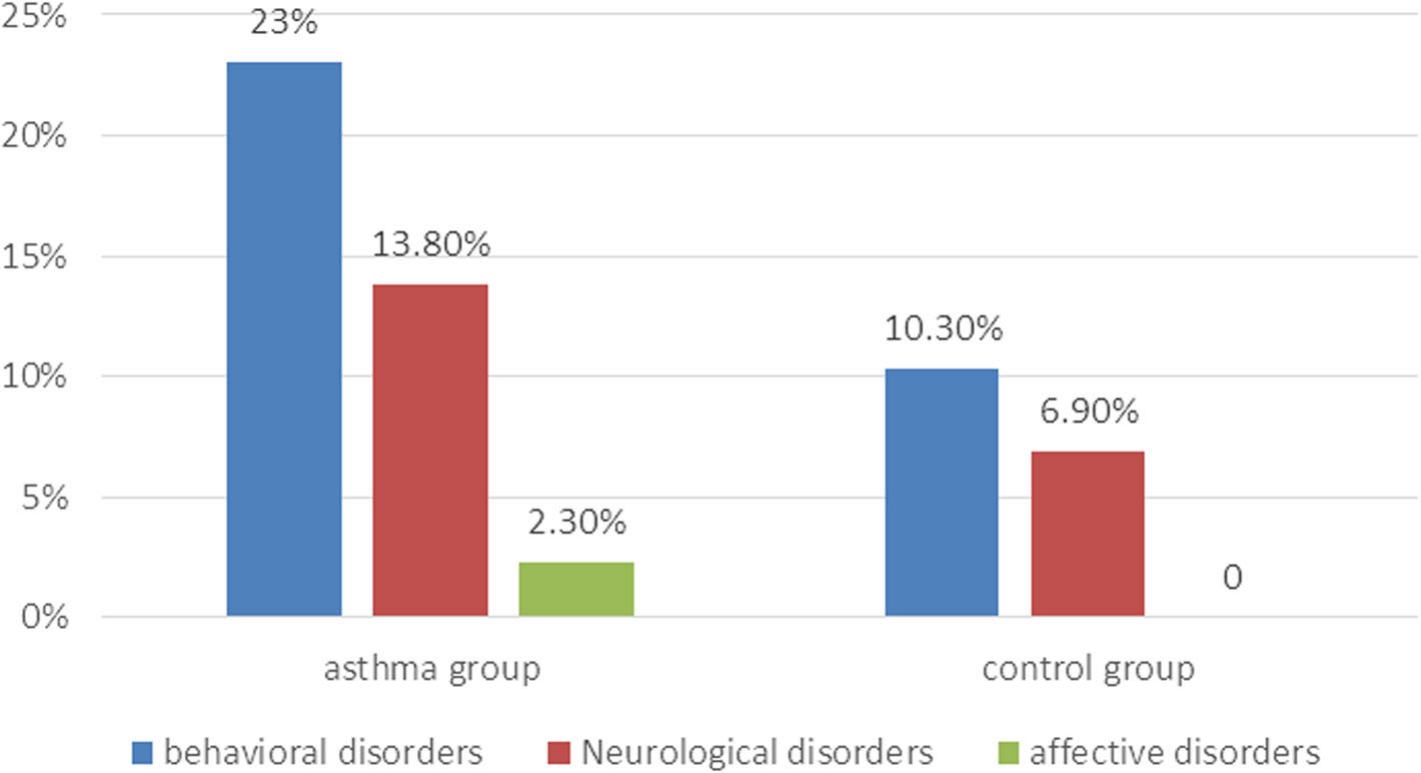
Comparison of neuropsychiatric comorbidities between asthma and control groups

All mental health conditions were classified into the following three types based on the DSM-IV diagnostic criteria: behavioural disorders, neurological disorders and affective disorders. The rates of these comorbidities were 23.0, 13.8, and 2.3% and 10.3, 6.9, and 0.0% in the asthma group and the control group, respectively (*P* < 0.05) (Fig. 1).

Overall, 26.4% (69/261) of the children with asthma had neuropsychiatric comorbidities, but only 7.2% (5/69) had been diagnosed previously and had received treatment, including one girl with depressive disorder and four boys with ADHD. In the control group, 14.6% (38/261) of children had neuropsychiatric comorbidities. Using 12 years of age as a cut-off, no significant differences were observed in the prevalence of comorbidities between children with asthma and control group children aged < 12 or ≥ 12 years.

### Analysis of asthma-related risk factors for neuropsychiatric comorbidities in children with asthma

We divided the asthma group into the following two groups based on the presence of comorbidities: asthma with neuropsychiatric comorbidities group (AWNC, *n* = 69) and asthma without neuropsychiatric comorbidities group (AONC, *n* = 192) (Table 3). No obvious age or gender differences were noted between the two groups.

**Table 3:** Univariate analysis of clinical characteristics of asthmatic children associated with neuropsychiatric comorbidities (*n*, %)

Variables	Category	AWNC (*n* = 69)	AONC (*n* = 192)	χ^2^	*P*
Onset of asthma	≤3 years	44(63.8)	105(54.7)	1.709	0.191
	>3 years	25(36.2)	87(45.3)		
Severity of asthma	Severe persistent	13(18.8)	13(6.8)	8.244	0.004
	Mild persistent	56(81.2)	179(93.2)		
Asthma control level	Poor control	40(58.0)	71(37.0)	9.151	0.002
	Good control	29(42.0)	121(63.0)		
Anti-asthmatic drug usage	ICS	16(23.2)	15(7.8)	10.04	0.001
	LTRA	4(5.8)	18(9.4)	0.471	0.493
	LTRA+ICS	10(14.5)	45(23.4)	1.933	0.164
	BA+ICS	6(8.7)	17(8.9)	0.043	0.835
	BA+ICS + LTRA	33(47.8)	97(50.5)	0.003	0.958
Anti-asthmatic therapy	Monotherapy	20(29.0)	33(17.2)	4.366	0.037
	Multidrug therapy	49(71.0)	159(82.8)		
Regular follow-up	Yes	40(58.0)	105(54.7)	0.222	0.638
	No	29(42.0)	87(45.3)		
Family history	Yes	18(26.1)	43(22.4)	0.386	0.534
	No	51(73.9)	149(77.6)		
Asthma attacks	≥1 time monthly	8(11.6)	20(10.4)	7.034	0.071
	≥ every 3 months, < 1 time monthly	32(46.4)	60(31.2)		
	≥ every 6 months, < 1 time every 3 months	16(23.2)	75(39.1)		
	No attack ≥1 year	13(18.8)	37(19.3)		
Duration	> 3 years	50(72.5)	90(46.9)	13.365	0.000
	≤3 years	19(27.5)	102(53.1)		

*AWNC* Asthma with neuropsychiatric comorbidities, *AONC* Asthma without neuropsychiatric comorbidities, *ICS* Inhaled corticosteroid, *LTRA* Leukotriene receptor agonists, *BA* Beta-2-agonists

Univariate analysis was used to identify potential risk factors associated with mental health conditions in asthma patients (Table 3). Four factors demonstrated significant differences between the asthma groups, including the severity of illness, level of disease control, duration of disease, and asthma drug use. Severe persistent asthma (OR = 3.077, 95% CI: 1.286–7.361), poor asthma control (OR = 2.005, 95% CI: 1.111–3.619), and duration of disease > 3 years (OR = 2.948, 95% CI: 1.580–5.502) were identified as independent asthma-related risk factors for mental health conditions in children with asthma (Table 4).

**Table 4:** Multivariate logistic regression analysis of clinical characteristics of asthmatic children associated with neuropsychiatric comorbidities

Variables	Parameter estimate	Standard error	Chi-square	*P* value	Odds ratio
Severe and persistent	1.124	0.445	6.379	0.012	3.077(1.286–7.361)
Poorly controlled	0.696	0.301	5.332	0.021	2.005(1.111–3.619)
Disease duration > 3 yr	1.081	0.318	11.534	0.001	2.948(1.580–5.502)
Multidrug therapy	0.467	0.353	1.752	0.186	1.595(0.799–3.183)

## Discussion

Chronic diseases are public health problems that affect population health, but previous studies have predominantly focused on adult chronic diseases. Currently, experts have begun to pay more attention to mental health in children with chronic diseases. Children with chronic diseases who experience states of long-term stress appear to exhibit various unusual psychological behaviours that affect their personalities and social interactions, have a serious impact on their quality of life and that of their families and potentially cause serious social problems [4]. However, despite ongoing investigation, the prevalence of and risk factors for mental health comorbidities in children with chronic diseases such as asthma remain unclear. Possible risk factors or associations suggested by the results of previous studies may include physical exercise, smoking, asthma medication use, underlying inflammation, and sleep [34–36]. Although many authors have pointed to associations between the stress of interpersonal conflicts or emotional distress and asthma exacerbation [16–21], fewer investigators suggest a specific pathogenetic mechanism such as mast cell activation [36] or the release of proinflammatory mediators [15, 23]. Still others suggest that psychosocial factors consistently impact airway pathophysiology in respiratory diseases such as asthma [35]. Airway response to psychological stimulation clearly needs further study.

The current study found that the prevalence of mental health conditions was 26.4% in children with asthma compared with 14.6% in the control group (*P* < 0.05). Additionally, the odds ratio for mental health conditions were significantly higher in the asthma group than in the control group (OR = 1.81; *p* < 0.05). In the asthma group, the most common mental health conditions (≥5%) were ADHD, ODD, and separation anxiety. In the control group, the most common conditions were ODD and ADHD, which were significantly less frequent than in the asthma group (*P* < 0.05). Independent asthma-related risk factors for mental health conditions were severe persistent asthma, poor asthma control, and having asthma > 3 years.

Children with asthma may be more likely to have mental health conditions. Findings from prior studies suggest that asthma severity is associated with an increased risk of mental health conditions, and children with severe asthma have rates of emotional disorders, anxiety disorders, ADHD, and ODD approximately 1–2 times higher than those with mild or moderate asthma [34]. Consistent with the findings of the present study, prior studies have suggested that asthma-related mental health conditions in early childhood may be associated with inadequate asthma control [37] and poor asthma control, particularly in patients prone to insomnia, anxiety, depression and other symptoms [27]. Good asthma control and reasonable asthma management appear to reduce the risk of anxiety and depression. Asthma cases with a long duration demonstrate a higher risk for mental health issues [38]. A previous study revealed that chronic diseases with a long duration may be associated with a higher risk of neuropsychiatric comorbidities [3]. In addition, prior studies have reported that the use of drugs to treat asthma, such as inhaled corticosteroids (ICs) and β2 agonists, may also be associated with progression to mental disorders [39]. The neurological side effects of these classic asthma-control drugs have been of widespread interest in recent years, particularly regarding the occurrence of adverse neurological events in children. These drugs may cause sleep disorders, cognitive dysfunction, neurodevelopmental delay, anxiety disorders, depression, and even suicidal ideation, suicidal behaviour, and delirium. However, even though these conditions may be more associated with the adverse effects of these drugs than with asthma itself, children undergoing asthma treatment may be more likely to show behaviour-related issues [40]. The results of the present study revealed that asthma medications may have affected children’s behaviour in univariate analysis. However, due to study designs and limited data, the current research has not found any specific asthma medications to be risk factors for neuropsychiatric comorbidities.

Our findings agree with previous studies that reported a higher incidence of mental health conditions in children with asthma than in the control group. In the present study, the most common behavioural mental health conditions in children with asthma were ADHD, ODD, and separation anxiety. One previous study reported that pre-schoolers treated for asthma in the prior 12 months had higher anxiety DSM-oriented scores (54.9; *P* = 0.024) [41]; those investigators also found a higher risk of sleep problems in preschool children treated for allergic rhinitis and an increased incidence of attention problems and ADHD scores in preschool children treated for atopic dermatitis. These findings suggest an association between the presence of allergic diseases and the development of psychological and behavioural problems.

The results of this study showed that severe persistent asthma, poor asthma control, and duration of disease > 3 years were independent risk factors for neuropsychiatric comorbidities in children. Previous reports suggest that increasing asthma symptom control, relieving asthma severity, and shortening the course of the disease may be associated with a reduced risk of neuropsychiatric comorbidities [42, 43].

Although severe factors associated with asthma and asthma treatment have been found to be risk factors for developing a mental health condition, evidence shows that mental health may also influence the development of asthma. The Coronary Artery Risk Development in Young Adults (CARDIA) study [26], conducted in the United States, evaluated possible associations between depressive symptoms and asthma. The study found that after a 20-year follow-up, depressive symptoms were a marker for the risk of developing adult-onset asthma. Another large study that utilized the National Health Insurance database of Taiwan to investigate adults newly diagnosed with major depressive disorder (MDD) (*N* = 30,169) found that the overall incidence of asthma was 1.94-fold higher in the MDD cohort than in the non-MDD cohort [27]. The study also found that in both cohorts, the incidence of asthma was higher in patients who were female, were older, had comorbidities, or used aspirin or beta-adrenergic receptor blockers. The authors concluded that adult patients with MDD were at higher risk of asthma than those without depression.

The findings of the present study and prior investigations indicate that mental health conditions may be prevalent in children with asthma. Often, these asthma-related conditions are underdiagnosed, possibly due to lack of a standardized assessments for psychiatric status in children with asthma. Favreau et al. [43] found that panic disorder and anxiety sensitivity are associated with worse asthma control and symptoms such as hyperventilation. Chun et al. [44] confirmed the relationship between asthma and mental health symptoms in a large nationally representative sample and suggested that the presence of any degree of poor mental health increased asthma risk, agreeing with other authors who suggest that mental health status may increase the risk of asthma [25, 26]. Therefore, guidelines for the early screening, diagnosis and treatment of neuropsychiatric comorbidities in children with asthma should be developed with the goal of improving long-term prognoses and management. The findings of the present study and previous studies suggest that clinicians/health professionals should be appropriately trained to diagnose mental health in children with asthma. The early diagnosis of and intervention in mental health conditions may help to improve patients’ quality of life and relieve the illness.

### Limitations

The results of the present study must be interpreted cautiously considering certain limitations. The study was a cross-sectional single-centre study, and the findings can only explain the characteristics of the current study sample. The study focus was to investigate whether asthma may increase the risk for mental health conditions in children 6 to 16 years old, but many variables may confound the findings, including the emotional reaction of the study population to being in the hospital, which may influence the outcomes of the MINI Kid tool. The risk factor analysis included only some factors such as medications and disease course related to asthma itself and did not include environmental and pathogenetic risk factors. In addition, the study did not evaluate psychosocial risk factor assessments or differentiate between children and adolescents or between affective and behavioural disorders. The study also used only one measure to explore mental health issues and did not assess factors that may increase the chance of developing a mental health condition. The study population was 6 to 16 years old and did not include younger children. Future prospective studies are needed among children, adolescents and young adults to help identify risk factors and physiological mechanisms behind the relationship between childhood asthma and mental health disorders.

## Conclusions

The prevalence of mental health conditions in asthmatic children is higher than that in non-asthmatic children, and severe persistent asthma, poor asthma control, and duration of disease > 3 years are independent risk factors for neuropsychiatric comorbidities. The study findings highlight the need for an integrated approach to asthma and mental health. The early diagnosis of and intervention in mental health disorders in children with asthma is necessary, and the provision of standardized asthma treatment may help to reduce the risk of neuropsychiatric comorbidities in children with asthma.

## Supplementary information

**Additional file 1:** This file included the “Introduction to the diagnostic sensitivity of the Chinese version of the MINI Kid for different neuropsychiatric diseases” and “The demographics and asthma clinical characteristics questionnaire”.

## Data Availability

The datasets used and/or analysed in the current study are available from the corresponding author upon reasonable request.

## Abbreviations

95% CI: 95% confidence interval
ADHD: Attention deficit/hyperactivity disorder
DSM-IV: Diagnostic and Statistical Manual of Mental Disorders, 4th edition
ICD-10: International Classification of Diseases, 10th Revision
MDE: Major depressive episode
MINI Kid: Mini-International Neuropsychiatric Interview for children and adolescents
ODD: Oppositional defiant disorder
OR: Odds ratio
SAD: Seasonal anxiety disorder
