# Supplementary material for: Evaluation of neuropsychiatric comorbidities and their clinical characteristics in Chinese children with asthma using the MINI kid tool
Source: BMC Pediatr. 2019 Nov 22;19:454. doi: 10.1186/s12887-019-1834-7 (PMC6873764; doi:10.1186/s12887-019-1834-7)
Supplement: Supplementary file 1 — Additional file 1: This file included the “Introduction to the diagnostic sensitivity of the Chinese version of the MINI Kid for different neuropsychiatric diseases” and “The demographics and asthma clinical characteristics questionnaire”. [file 12887_2019_1834_MOESM1_ESM.docx]

1. **The diagnostic sensitivity of the Chinese version of the MINI Kid for different neuropsychiatric diseases and the**

The Chinese questionnaire was most sensitive to the diagnosis of ADHD (32.0%), oppositional defiant disorder (30.9%), conduct disorder (78.6%), children's affective and mood disorders (66.3%), tic disorder (58.5%), psychotic disorders (93.6%), and posttraumatic stress disorder (79.2%). The questionnaire's diagnostic specificity toward these various conditions was ADHD (95.8%), oppositional defiant disorder (94.5%), conduct disorder (94.5%), children's affective and mood disorders (66.8%), tic disorder (98.8%), psychotic disorders (98.6%), and posttraumatic stress disorder (92.7%). Combined diagnosis was performed on the basis of the results of the two versions for the 319 children for whom the children's version and parents' version had been fully completed, and the results indicated that diagnostic sensitivity was ADHD (86.5%), oppositional defiant disorder (83.5%), conduct disorder (92.9%), children's affective and mood disorders (73.7%), tic disorder (73.6%), psychotic disorders (97.9%), while diagnostic specificity was ADHD (95.9%), oppositional defiant disorder (88.8%), conduct disorder (91.1%), children's affective disorders and mood disorders (66.7%), tic disorder (98.5%), and psychotic disorders (98. 5%).

**2. The demographics and asthma clinical characteristics questionnaire**

1. Name, sex, date of birth
2. Place of residence: (1) Urban (2) Suburban or rural
3. Ethnicity: (1) Han (2) Ethnic minority
4. Educational level of chief caregiver: (1) High school and above(2)Below high school
5. Monthly household income: (1) < RMB 2000/month (2) RMB 2000-5000/month (3)＞RMB 5000/month
6. Whether the only child:(1)Yes (2)No
7. Whether the left-behind child:(1)Yes (2)No
8. Parents Relationship: (1)Harmonious (2)Not harmonious
9. Family structure:(1)Single-parent family (2)Two-parent family (3)Three generations live under the same roof
10. Family history of psychiatric:(1)Yes (2)No
11. Child-raising method:(1) Authoritative(2)Arbitrary(3)Permissive(4)Negligent
12. Whether living in school: (1)Yes (2)No
13. Health insurance: (1)Yes (2)No

***Asthma clinical characteristics questionnaire***

1. Onset of asthma:(1) ≤3 years (2) ＞3 years
2. Severity of asthma: (1) Severe persistent (2) Mild persistent
3. Asthma control level: (1)Poor control (2)Good control
4. Anti-asthmatic drug usage:(1)ICS (2)LTRA (3)LTRA+ICS(4) BA+ICS

(5)BA+ICS+LTRA

1. Drug kinds:(1)Monotherapy(2)Multidrug therapy
2. Receives regular follow-ups: (1) Yes, (2) No
3. Family history of asthma: (1) Yes, (2) No
4. Frequency of asthma attacks: (1) ≥1 per month; (2) every 3 months ≥1 time and < 1 per month; (3) every 6 months ≥1 time and < 1 each 3 month; (4) no attacks in one year
5. Duration of asthma: (1)＞3 years（2）≤3 years

**Note:** The left-behind child mean that the children whose parents go out to work while they stay in the countryside. They usually live with their grandparents and even other relatives and friends of their parents. With the further development of China's reform and opening-up, which lead to the social phenomenon of left-behind children.
